# Peristomal skin complications in ileostomy and colostomy patients: a systematic literature review

**DOI:** 10.1093/eurpub/ckac131.575

**Published:** 2022-10-25

**Authors:** F D'Ambrosio, C Pappalardo, A Scardigno, A Maida, R Ricciardi, GE Calabrò

**Affiliations:** 1 Section of Hygiene, University Department of Public Health, Università Cattolica del Sacro Cuore, Rome, Italy; 2 VIHTALI, Spin-Off of Università Cattolica del Sacro Cuore, Rome, Italy

## Abstract

**Background:**

Peristomal skin complications (PSCs) are one of the main post-operative complications of ostomy surgery. They have a considerable impact on patients’ quality of life and represent a challenge for healthcare professionals involved in their management. The majority of PSCs is preventable and costly. Knowing their burden could guide decision makers on the ostomy patients’ management who are predominantly cancer and chronic bowel disease patients. Thus, the aim of this study was to summarize existing literature regarding the clinical-epidemiological burden of PSCs in ostomy patients.

**Methods:**

A systematic literature review was performed querying three database (PubMed, Scopus, Web of Science) from January 2012 to February 2022. It included studies in English language and focused on the clinical and epidemiological burden of PSCs in the adult patients with ileostomy and colostomy.

**Results:**

Overall, 35 studies were considered. Epidemiological data on PSCs were not systematically collected in the available literature. The principal underlying disease requiring the ostomy surgery were rectal, colon and gynaecological cancers, inflammatory bowel diseases, diverticulitis, occlusion and intestinal perforation. It was described a range of PSCs from 11% to 45%. The PSCs were most commonly erythema, papules, erosion, ulceration and vesciculation. Skin complications increased the average number of hospitalization days and of hospital readmission within 120-day following surgery.

**Conclusions:**

The data on PSCs are still limited. Estimating the PSCs burden could support healthcare professionals and decision makers called upon to identify the most appropriate responses to patients’ health needs. The management of these complications plays a vital role to improve patient’s quality of life and a multidisciplinary approach with the active involvement of stomatherapist, surgeon and dermatologist is critical. Furthermore, a better patient education and empowerment is needed.

**Key messages:**

• Developing a multidisciplinary approach to managing PSCs is essential in order to provide the best treatment possible and the best outcomes for patients.

• Further studies should be conducted in order to better define the clinical-epidemiological burden of ileo- and colostomies and to support better health planning.

